# Registered nurses’ clinical reasoning in home-based care: a retrospective think-aloud design – the DECIDE project

**DOI:** 10.1016/j.ijnsa.2026.100621

**Published:** 2026-07-09

**Authors:** Ida Røed Flyum, Veronica Pavedahl, Anna Josse Eklund, Gunilla Borglin

**Affiliations:** a Lovisenberg Diaconal University College, Department of Nursing, Lovisenberggata 15B, NO-0456, Oslo, Norway; b Karlstad University, Institute of Health Sciences, Department of Nursing, Universitetsgatan 2, 651 88, Karlstad, Sweden

**Keywords:** Clinical reasoning, Registered nurses, Home-based care, Decision-making, Think-aloud method, Qualitative analysis

## Abstract

**Background:**

Clinical reasoning in nursing is described as complex, context-dependent, and difficult to articulate. Despite its recognised importance, it is often examined through narrative accounts or inferred from decision outcomes, offering limited insight into how reasoning is articulated and organised in practice. This has limited our understanding of the structure of reasoning, particularly in complex and dynamic settings, and underscores the need for approaches that make this structure explicit and open to systematic analysis.

**Aim:**

To explore and describe how registered nurses retrospectively explain and structure their clinical reasoning in relation to observed care situations in home-based care.

**Methods:**

A retrospective think-aloud design was used. Nineteen transcripts from registered nurses were analysed using a rule-based modified clinical protocol analysis, with AI-assisted procedures to identify clinical domains, operator configurations, and cross-case structures.

**Results:**

Clinical reasoning was consistently organised around recurring patterns of observation and evaluation, often extended into planning in more complex or risk-oriented situations. Reasoning was characterised by non-linear structures combining diagnostic and procedural orientations. Different clinical domains were associated with distinct reasoning structures, including verification-based processes in procedural care and evaluation–planning processes in autonomy- and support-focused situations.

**Conclusion:**

Registered nurses’ clinical reasoning in home-based care can be described as structured, multi-layered, and contextually adaptive, rather than strictly linear. The findings suggest that reasoning can be systematically described across cases, making reasoning structures visible and comparable.

**Implications:**

This study offers a basis for describing and discussing clinical reasoning more systematically in nursing education, supervision, and future research.

**What is already known**

• Clinical reasoning is complex and central to nursing practice
• Reasoning is often described as tacit, experience-based and difficult to articulate
• How reasoning is structured and what influences it is less well understood

Alt-text: Unlabelled box dummy alt text

**What this paper adds**

• Recurring reasoning structures were identified across transcripts and clinical domains
• This study shows how retrospectively articulated reasoning can be described systematically across cases
• More complex and risk-related care situations were associated with expanded reasoning structures, often including planning

Alt-text: Unlabelled box dummy alt text

## 1 Introduction

How registered nurses (RNs) reason in practice is central to understanding the cognitive dimension of their professional scope of practice. Although reasoning is widely recognised as integral to nursing work, how it is structured and articulated in clinical situations remains insufficiently described. Within the operationalisation of nurses’ scope of practice as their responsibility (role), activities and tasks (function), and decision-making capability (cf. International Council of Nurses [ICN], 2025; Malone and Cliffe, 2018; Royal College of Nursing [RCN], 2023), the latter highlights the importance of decision-oriented professional reasoning. Central to this capability is clinical reasoning, which underpins how RNs interpret patient situations, form clinical judgments, and decide on appropriate actions. A range of related terms are used in the literature to describe nurses’ cognitive processes, including clinical reasoning, clinical judgment, decision-making, and critical thinking. These concepts have often been used interchangeably since at least the 1980s (e.g., Benner, 1984; Tanner, 2006; Connor et al., 2023), contributing to ongoing conceptual ambiguity. In the present study, clinical reasoning was selected as the focal concept because the aim was to examine how RNs retrospectively articulated and structured their reasoning in relation to observed care situations, rather than to evaluate the correctness of judgments, decisions, or outcomes. Despite this conceptual overlap, such cognitive processes are widely recognised as foundational to RNs’ competence, underpinning all aspects of nursing practice, including decisions about what not to do and what to delegate (Ranta and Kaunonen, 2024; Thompson and Stapley, 2011).

Clear understanding of these processes is particularly important in home-based care, where RNs’ responsibilities continue to expand alongside increasing overlap with other professionals (Flyum et al., 2026). In these settings, RNs are often the first to identify subtle changes in patients’ conditions, requiring rapid and context-sensitive reasoning. Empirical evidence demonstrates that nurses’ competence in clinical judgment and decision-making is associated with patient outcomes, with failures in these processes linked to adverse events and unfinished care (Doyon and Raymond, 2025). RNs’ reasoning in practice is closely connected to how they interpret situations, prioritise concerns, and formulate nursing actions within their scope of responsibilities.

Clinical reasoning, judgment, and decision-making do not occur in isolation but are shaped by contextual modulators. These include fluctuating patient needs, brief and intermittent care encounters, and expectations of professional autonomy (Brenne et al., 2022; Flyum et al., 2026). In addition, increasing complexity in older people’s care needs, including frailty and functional limitations, further intensifies the cognitive demands placed on RNs (Vermeiren et al., 2016). In home-based care, where RNs often work alone and must respond to evolving patient situations without immediate support, clinical reasoning becomes particularly critical.

Despite its centrality, clinical reasoning has frequently been described as a “black box,” as it is difficult to observe directly and challenging for practitioners to articulate. This has contributed to a field characterised by conceptual ambiguity and limited empirical insight into how reasoning is enacted in practice. Recent literature reviews highlight that research on clinical reasoning in nursing remains predominantly focused on educational contexts, with limited empirical understanding of how reasoning is retrospectively articulated and structurally organised in clinical situations (Hurson et al., 2026; Neethling and Roets, 2025).

Clinical reasoning is not always explicit or linear; rather, it often involves tacit and experience-based processes shaped by context, meaning that nurses may “know” what to do without fully articulating their reasoning (Benner, 1984; Tanner, 2006). While this perspective captures the complexity of nursing practice, it also contributes to the continued characterisation of clinical reasoning as difficult to examine systematically. Consequently, and despite that important conceptual distinctions have been proposed (Banning, 2008; Simmons, 2010), there is limited empirical understanding of how clinical reasoning is structured and organised in clinical practice, how it unfolds across different patient situations, and how it is articulated by RNs themselves.

Think-aloud technique has been used to explore clinical reasoning in home healthcare nursing, providing insight into how RNs articulate and describe their reasoning in relation to patient care (Johnsen et al., 2016; Sockolow et al., 2021; Smet et al., 2024). Across these studies, clinical reasoning is described as highly context-dependent and shaped by patient-specific knowledge, with RNs often relying on recognising deviations from patients’ usual condition and integrating environmental and relational factors into their assessments. However, previous research has primarily focused on specific clinical care situations or single-case analyses and has typically applied qualitative or protocol-based approaches. There remains limited research systematically examining how clinical reasoning is structured across multiple cases and clinical care situations within home-based care, using traceable analytic approaches that retain close adherence to RNs’ own verbalised accounts. This gap is particularly relevant in home-based care, where complex and evolving patient needs require continuous interpretation, prioritisation, and adaptation. Accordingly, this study was based on the assumption that the specific context of home-based care may influence how clinical reasoning is articulated and structured in practice. To address this gap, this study focuses on clinical reasoning as the central process underpinning nursing practice. This study aimed to explore and describe how registered nurses retrospectively explained and structured their clinical reasoning in practice in relation to observed care situations in home-based care.

## 2 Methods

### 2.1 Study design

In this study, an exploratory and descriptive qualitative design (Sandelowski, 2000; Stebbins, 2008) was used to gain insight into RNs clinical reasoning as a complex phenomenon. The study was exploratory in seeking to examine how RNs articulated and structured their clinical reasoning in home-based care, and descriptive in its aim to systematically characterise the reasoning structures identified across cases. Data was collected using the retrospective think-aloud (TA) technique (Ericsson and Simon, 1993). Transcripts from the TA conversations were analysed using an AI-assisted modified clinical protocol analysis (MCPA). The Standards for Reporting Qualitative Research (SRQR) was followed in the reporting of this study (O’Brien et al., 2014).

### 2.2 Study setting, recruitment strategies, and sample

This study was conducted in three home-based nursing teams within a larger Norwegian municipal home healthcare service. Home-based nursing care provided by RNs is, in Norway, largely delivered to older people, many of whom have complex care needs (Saunes et al., 2020). Norwegian RNs complete a three-year bachelor’s degree, which confers the academic credentials and professional licensure required for RN practice.

The RNs included in this study, and the older care recipients they cared for during data collection, were part of a larger research project, DECIDE, involving two steps of data collection. The first step consisted of structured observations of care situations followed by retrospective TA, while the second step (not reported in this paper) involved additional data collection within the broader project. Although both RNs and older care recipients were recruited within the larger DECIDE project, the present study analyses only RNs’ retrospective TA transcripts. Care recipients formed part of the observed care context but were not treated as primary analytic units in this study. The data analysed and reported in this study were collected as part of the first step.

RNs affiliated with the three included nursing teams were recruited using purposive sampling (Patton, 2015). RNs were evenly distributed across the included teams. Inclusion criteria were permanent or long-term temporary employment, direct involvement in nursing care for older people with complex needs, and proficiency in Norwegian. Care recipients were recruited as part of the broader project context. Recruitment was conducted in collaboration with team leaders and professional development RNs. For further details on the setting and recruitment, see Flyum et al. (2026).

### 2.3 Data collection

Retrospective TA (Ericsson and Simon, 1993; van den Haak and De Jong, 2003) was used as the data collection method. The TA technique is described as well suited for the investigation of complex cognitive processes in real-world settings, including clinical reasoning (Laukvik et al., 2023; Johnsen et al., 2016). Retrospective TA was considered appropriate as the aim was to explore how RNs describe and reason about clinically situated cognitive processes rather than to capture moment-to-moment information processing (van den Haak et al, 2003). Immediately before each retrospective TA, the researcher conducted a structured observation of the RNs’ care encounter using a predefined observation protocol. These observations were not included as data in the present paper; rather, they functioned as situational anchors during the subsequent TA. The TA conversations were conducted immediately after the structured observations to keep the cognitive processes current in participants’ memory and reduce post hoc rationalisation. The TA were audio-recorded and transcribed using intelligent speech recognition technology (Eftekhari, 2024), followed by manual verification to ensure accuracy.

### 2.4 Data analysis

**Fig. 1:**
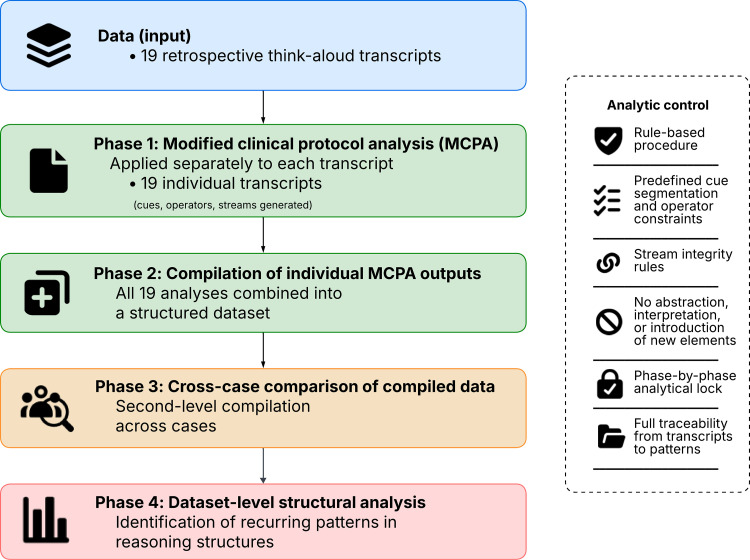
Rule-based analytic pipeline for clinical reasoning analysis.

The data analysis was conducted in a structured four-step process using a modified clinical protocol analysis (MCPA) supported by a rule-based analytic framework, as illustrated in Fig. 1.

Nineteen retrospective think-aloud transcripts were analysed in a sequential four-step process. Individual MCPA analyses were compiled, compared across cases, and aggregated at dataset level. Predefined rules ensured full traceability to the original transcript data.

In Step 1, data were analysed using the MCPA, in which verbal accounts were segmented into discrete cues, assigned predefined cognitive and assertion operators, and organised into object-specific reasoning streams. Each cue was assigned one cognitive operator and one aligned assertion operator according to predefined compatibility rules. The cognitive operators captured the functional orientation of the reasoning statement (Observe, Verify, Evaluate, Connect, Conclude, Plan), while the assertion operators described its linguistic form (Declarative, Evaluative, Significative, Inferential, Goal-oriented). One cue could receive only one operator pair. To further illustrate Step 1 of the analytic process shown in Fig. 1, Table 1 presents an example from RN13 showing how transcript excerpts were segmented into cues, assigned assertion–cognitive operator pairs, and organised into a reasoning stream within a clinical domain.

**Table 1:** Illustrative example of cue coding, operator assignment, and reasoning stream construction.

Transcript excerpt (RN13)	Cue code	Assertion operator	Cognitive operator	Reasoning statement	Reasoning stream and clinical domain	Dominant stream operator pattern
“I think he should be on oxygen all the time.”	Oxygen therapy	Declarative	Observe	Therapy noted		
“But he keeps taking it off.”	Oxygen non-adherence	Evaluative	Evaluate	Non-adherence assessed		
“He becomes short of breath when he tries to do things himself.”	Dyspnea	Declarative	Observe	Dyspnea noted	Respiratory assessment	Observe/Evaluate
“Today, I also noticed rattling in his breathing.”	Respiratory sound	Declarative	Observe	Sound noted		
“It was more than usual.”	Deviation respiratory	Evaluative	Evaluate	Deviation assessed		

**Note.** This example illustrates how transcript excerpts were segmented into cues, assigned predefined assertion–cognitive operator pairs, and organised into a reasoning stream within a clinical domain during Step 1 of the analytic process illustrated in Fig. 1.

Reasoning streams were constructed only from coded cues and were restricted to a single clinical focus within a domain and a single reasoning direction. These streams formed the basis for identifying recurring reasoning structures within and across cases. In Step 2, the outputs from the individual MCPA analyses were compiled into a standardised structural format to ensure consistency across transcripts while maintaining full traceability to the original data. In Step 3, a structured cross-case comparison was conducted on the compiled dataset to identify patterns across cases in clinical domains, reasoning streams, and operator configurations. This step focused on comparing structural features of reasoning without reinterpretation of the underlying data. In Step 4, dataset-level analysis was performed based on the cross-case output to identify recurring patterns, variation, and relationships between clinical domains and reasoning structures across the dataset. The analysis was conducted sequentially, with all steps governed by predefined rules to ensure consistency and maintain full traceability to the original transcript data. No abstraction beyond the transcript data was introduced at any stage of the analysis. Quality control was applied throughout all analytic steps through continuous checking of cue segmentation, operator assignment, and reasoning stream construction to ensure adherence to the predefined analytic rules.

The analytic approach was designed to maintain close adherence to the original transcript data while minimising interpretive drift through predefined rules and operator assignments. This enabled a transparent and structured link between raw data and higher-level representations of reasoning. The approach draws on principles of protocol analysis and clinical protocol analysis, particularly regarding the systematic structuring of verbal data and the importance of traceability (Ericsson and Simon, 1993; Nusair et al., 2019), while extending these approaches through a more explicitly rule-based and structured analytic framework.

The analysis was conducted iteratively, with continuous checking of operator consistency and stream construction to ensure adherence to the analytic rules. All outputs were reviewed and validated by the researchers. Within the rule-based analytic framework, the AI functioned as a technical support tool to assist with cue identification, operator assignment, and stream organisation. It did not generate themes, interpretations, or explanatory categories. In addition, a subset of transcripts (n = 10) was independently analysed manually and compared with the AI-assisted outputs. The AI-assisted (last author) and manual analyses (first author) were conducted in parallel by different members of the research team, allowing independent analytic processes to be compared. In addition, the remaining authors (second and third authors) contributed as continuous critical reviewers and conducted independent checks of selected transcripts to support analytical rigour. The AI did not replace researcher judgement at any stage of the analysis.

### 2.5 Rigour and trustworthiness

Rigour and transparency were ensured through a structured, rule-based analytic framework applied consistently across all transcripts. The analysis was guided by predefined rules to maintain close adherence to the original transcript data and minimise interpretive drift. The structured nature of the approach supported consistency across cases while enabling comparison across the analytic process. Researcher control was maintained throughout the process through continuous review and validation of all analytic outputs. A subset of transcripts (n = 10) was independently analysed manually and compared with AI-assisted outputs to ensure consistency and adherence to the analytic protocol. The analytic procedure was developed iteratively through pilot testing and refinement prior to full data analysis. An audit trail was maintained across all analytic steps, enabling full traceability from transcript data to higher-level representations of reasoning. Quality control procedures were applied throughout the analysis to ensure consistent application of cue segmentation, operator assignment, and stream construction. These procedures included independent manual and AI-assisted analyses, comparison of analytic outputs, independent checking of selected transcripts by additional members of the research team and review of analytic discrepancies to ensure adherence to the predefined analytic rules and minimise potential analytical bias.

### 2.6 Ethical considerations

This study was reviewed by the Regional Committees for Medical and Health Research Ethics, the Norwegian Agency for Shared Services in Education and Research (IDs 733476 and 349256), the Research Ethics Committee at Karlstad University, and the Swedish Ethical Review Authority (IDs C2024/636 and 2025-00816-01). The Regional Committees in Norway and the Swedish Ethical Review Authority determined that the project was not within their remit. All participants provided written informed consent and received both verbal and written information. Care recipients’ capacity to provide informed consent was assessed by the RNs, and proxy consent was obtained when required following a benefit–risk assessment. For the AI-assisted analysis, all transcripts were pseudonymised prior to analysis to ensure that no personally identifiable information was included. A temporary AI-assisted analytical workflow was used and deleted after completion of the analysis.

## 3 Results

Nineteen transcripts of retrospective TA, exploring how RNs explain and structure their clinical reasoning in home-based care, were analysed using a MCPA. Of the nineteen RNs, fifteen were female and four were male. The mean number of years of experience in home-based care was approximately ten years, with a range of one to thirty years. The analysis identified both variation and recurring reasoning structures in RNs’ reasoning across cases, and in relation to distinct clinical domains. The main results concern three related levels: the clinical domains represented across transcripts, the recurring reasoning structures identified within and across cases, and the relationships between clinical domains and reasoning structures at dataset level.

### 3.1 Clinical domains represented across cases

The dataset represents a broad range of clinical domains, reflecting the main areas of care around which the RNs’ reasoning was articulated. In this study, clinical domains refer to the main areas of care that shaped each observed care situation and around which the RNs’ reasoning was articulated. All cases involved multiple clinical domains, ranging from four to six clinical domains per case. Functional assessment appeared across the majority of cases and was consistently present alongside other clinical domains such as medication management, nutrition, wound care, respiratory assessment, compression therapy, and risk-related areas including fall risk and infection. Additional clinical domains included psychosocial and behavioural aspects (i.e. behavioural observation, risk and adaption), self-care capacity, and procedure-based care such as stoma care and dialysis. This distribution suggests that reasoning was embedded in multifaceted care situations in which multiple clinical domains were often addressed simultaneously rather than in isolation (Table 2).

**Table 2:** Overview of clinical domains represented across transcripts.

RN case^a^	Clinical domains
RN1	Stoma care; symptom assessment; psychosocial assessment; functional assessment; nutrition
RN2	Wound care; infection assessment; healing process; functional assessment; skin condition
RN4	Medication management; functional assessment; frailty assessment; behavioural observation
RN5	Respiratory assessment; circulatory assessment; functional assessment; medication management; nutrition
RN6	Routine care; infection assessment; skin assessment; functional assessment; catheter-related risk
RN7	Stoma care; activity/mobility; pain assessment; medication management; nutrition; hygiene
RN8	Functional assessment; nutrition; psychosocial assessment; hygiene management; fall risk
RN9	Stoma care; wound care; skin integrity; functional assessment; fall risk
RN10	Functional assessment; medication management; nutrition; stoma care; self-care capacity; ergonomic risk
RN11	Functional assessment; cognitive assessment; medication management; rehabilitation/mobility; observation; care planning
RN12	Functional assessment; pain assessment; respiratory assessment; self-care capacity; behavoural risk; care planning
RN13	Medication management; respiratory assessment; functional assessment; self-care capacity; care planning; observation
RN14	Functional assessment; self-care capacity; cognitive status; catheter care; nutrition
RN15	Wound care; nutrition; skin integrity; pain/irritation; functional assessment
RN16	Medication management; communication; functional assessment; fall risk; behavioural adaptation
RN17	Dialysis care; medication management; functional assessment; fall risk; infection prevention
RN18	Compression therapy; nutrition; pain assessment; functional assessment; assistive device planning
RN19	Dialysis care; respiratory assessment; pain assessment; functional assessment; fall risk
RN20	Functional assessment; compression therapy; diabetes monitoring; skin integrity; communication

a Registered nurses (RNs) are equally distributed across three teams; therefore, identification codes are not presented in numerical order. The identification numbers reflect the original participant coding within the larger study and are not consecutive; the present dataset comprises 19 transcripts.

### 3.2 Recurring structures of reasoning

Several recurring reasoning structures were identified across the cases, ranging from the most fundamental – observation and evaluation – to more complex configurations incorporating verification, conclusion, and planning. The number and structural complexity of reasoning structures per case varied, ranging from 5 to 11. The analysis further showed that the RNs’ reasoning was non-linear, in the sense that operator sequences did not consistently follow a single forward progression but frequently involved returns, shifts, and overlapping or parallel reasoning streams. For example, some sequences began with evaluation and planning before shifting back to observation and verification. These findings show that RNs’ reasoning followed identifiable structures while remaining flexible in how these structures were enacted. For example, one RN simultaneously attended to multiple clinical domains and moved between them during the same reasoning sequence: “When I entered, I was thinking about hygiene, nutrition, and activity. Because of time constraints, I had to prioritise, but I still asked about eating, physical condition, and daily functioning in order to get an overview of whether his basic needs were being met.” (RN7). This account illustrates how reasoning frequently moved across several concurrent clinical domains rather than following a single sequential pathway.

### 3.3 Variation in reasoning across cases

The analysis showed that the number and complexity of RNs’ reasoning structures also varied across transcripts. Some cases were characterised by relatively compact reasoning structures based on observation and evaluation, whereas others involved multiple concurrent reasoning structures across different clinical domains. More complex cases, particularly those involving instability, multiple risks, or ongoing interventions, demonstrated expanded reasoning structures with a stronger emphasis on planning and follow-up. For example, one RN described simultaneously weighing pain, alcohol use, functional decline, autonomy, pressure injury risk, and future care needs: “There are many considerations to take into account.” (RN12). Throughout the account, the RN moved between concerns related to symptom management, patient preferences, long-term care needs, and potential risks, illustrating the expanded and multi-layered nature of reasoning in more complex situations. In contrast, cases centred on routine monitoring or stable conditions more often relied on observation – evaluation or observation – evaluation – conclusion reasoning structures. This variation is illustrated through two contrasting cases. The two cases below are presented as structural illustrations of variation identified in the full dataset, not as representative typologies in themselves.

An illustrative example from a more routine case demonstrated a relatively contained reasoning structure. Reasoning was primarily organised around observation and evaluation, with limited extension into planning. Initial orientation was guided by unfamiliarity and reliance on the care plan, followed by assessment of self-care, functional and cognitive status, and catheter condition. For example, even within a relatively contained case, reasoning could shift from observation and evaluation to immediate action, as illustrated in the following account: “I think he should be on oxygen all the time, but he keeps taking it off. During care, he becomes short of breath when he tries to do things himself, which is why I do it for him. Today, I also noticed rattling in his breathing, so I did not ask him to do anything—I managed the care myself.” (RN13). Reasoning within these clinical domains was largely evaluative, focusing on interpreting observed cues rather than initiating intervention. Planning occurred only occasionally, for example in relation to potential support needs or food preparation. Overall, the reasoning remained structured but compact, with few transitions into action-oriented processes. In contrast, a more complex case demonstrated an expanded and multi-layered reasoning structure. Reasoning was organised across multiple concurrent reasoning structures, including procedural control, risk assessment, functional evaluation, and infection awareness. Observation and evaluation were frequently extended into planning, particularly in relation to risk management, procedural execution, and follow-up care. Procedural reasoning involved repeated verification and control steps, such as checking equipment and medication, while risk-oriented reasoning integrated observations of instability, physiological changes, and potential deterioration into planning of further actions. In addition, relational and contextual information was incorporated into ongoing assessment of cognitive and clinical status. Reasoning in this case was dynamic and distributed across clinical domains, with frequent transitions between assessment, verification, and action-oriented processes. Together, these examples illustrate how reasoning structures ranged from contained, assessment-focused forms to expanded, multi-domain and action-oriented forms, depending on the clinical situation and level of complexity.

### 3.4 Relationships between clinical domains and reasoning structures

The analysis further explored relationships between clinical domains and the reasoning structures enacted by the RNs. Functional assessment, the most frequently represented clinical domain across cases, was the only clinical domain in which reasoning consistently included conclusion, typically preceded by observation and evaluation, and was characterised by a more conclusive reasoning process. Primarily procedural clinical domains, such as stoma care and medication management, were characterised by reasoning structures incorporating observation, verification, and planning. One RN described actively verifying possible signs of infection before reaching a conclusion: “I looked for signs of infection—whether the wound was draining, whether there was redness, swelling, or pain. None of those signs were present.” (RN2). This example illustrates how observation was followed by verification before further evaluation and planning. Clinical domains involving diagnostic and therapeutic interventions, such as respiratory assessment and compression therapy, also included verification; however, the reasoning structure differed, typically following observation, verification, and evaluation. Clinical domains centred on function and autonomy were characterised by reasoning structures combining observation, evaluation, and planning. Table 3 summarises recurring clinical domain–reasoning relationships identified through cross-case comparison of stream structures and dominant operator patterns. The table does not introduce new analytic categories; it aggregates recurrent structural configurations already established through the within-case and cross-case analyses.

**Table 3:** Relationships between clinical domains and recurring reasoning structures.

Clinical domain	Reasoning structure	Dominant operator pattern
Functional assessment	Comparison of current status with baseline and observation-based judgement of abilities and needs	Observe → Evaluate (± Conclude)
Wound care	Assessment of wound status linked to treatment planning, healing evaluation, and prevention of complications	Observe → Evaluate → Plan
Medication management	Monitoring and control of medication routines with verification and adjustment of administration	Observe → Verify → Plan / Evaluate → Plan
Respiratory assessment	Monitoring of symptoms and comparison to baseline to detect change or deterioration	Observe → Evaluate
Nutrition	Assessment of intake and nutritional status linked to planning of support or intervention	Evaluate → Plan
Fall risk	Evaluation of instability, history, and functional limitations linked to preventive planning	Observe → Evaluate → Plan
Stoma care	Procedural management combined with assessment of condition and function of the stoma	Observe → Verify → Plan
Dialysis care	Procedural organisation and monitoring of treatment combined with risk awareness and adjustment	Observe → Evaluate → Plan
Compression therapy	Evaluation of fit, effectiveness, and validity of intervention with verification and follow-up	Evaluate → Verify → Observe
Psychosocial behavioural	Evaluation of patient response, motivation, and coping, sometimes linked to supportive planning	Evaluate (± Plan)

Procedure-bound clinical domains were more frequently associated with verification and planning-oriented reasoning structures. Clinical domains related to function, autonomy, and self-care capacity were more commonly associated with evaluation and adaptive planning. Risk-related clinical domains were typically associated with observation – evaluation – planning structures. The findings show that most reasoning structures, regardless of clinical domain, began with observation; however, the subsequent unfolding of reasoning was shaped by the clinical domain in focus. The analysis suggests a tendency among the RNs to adopt a hybrid form of reasoning, integrating both more observation-driven and more evaluation- and planning-oriented operator patterns.

### 3.5 Overall characteristics of clinical reasoning

Taken together, the findings show that clinical reasoning in home-based nursing care is structured, multi-layered, and contextually adaptive. Reasoning was most often characterised by combined and flexible patterns rather than isolated or purely linear processes. Diagnostic and procedural orientations were frequently intertwined, and reasoning structures varied depending on the clinical situation, the type of care required, and the care recipient’s functional status.

## 4 Discussion

This study aimed to explore and describe how RNs retrospectively structure their clinical reasoning in relation to observed care situations in home-based care. Using a rule-based analytical approach, we identified both recurring reasoning structures and systematic variations in how RNs’ reasoning unfolded across cases and clinical domains. Our findings indicate that clinical reasoning can be described as structured, multi-layered, and contextually sensitive, rather than linear. The structural complexity of the RNs’ reasoning structures varied across clinical domains, showing that reasoning was both patterned and contextually sensitive. These findings will be discussed below, both in relation to the existing literature and the potential implications for the understanding of RNs’ clinical reasoning in home-based care.

Our findings showed that the complexity of RNs’ reasoning structures varied systematically in relation to the clinical domain. RNs’ reasoning extended across multiple distinct clinical domains, such as functional assessment, wound care, dialysis, and compression therapy, mirroring the variations RNs meet in practice. The descriptions of RNs’ reasoning suggest that multiple clinical domains were addressed simultaneously and in parallel, rather than in isolation. Although most reasoning structures began with observation, subsequent reasoning varied across clinical domains, ranging from more conclusive structures to verification- and planning-oriented configurations, as well as structures characterised by evaluation and adaptive planning. These findings are consistent with previous descriptions of clinical reasoning as context-sensitive, situated, and adaptive (Tanner, 2006; Hoffman, 2007). Importantly, our findings suggest that such reasoning follows recurring structures that can be examined systematically to increase understanding and potentially move beyond individual, narrative, and experience-based descriptions.

The consistently identified non-linear structures of RNs’ reasoning suggest that their retrospectively articulated reasoning structures did not conform to a simple stepwise progression, neither linear nor circular. Instead, their accounts show how they move back and forth between observing, evaluating, verifying, and planning, or engage in multiple functions simultaneously within parallel structures. Importantly, this non-linearity was not uniform but varied systematically across clinical domains. In domains such as functional assessment, reasoning more often remained evaluative and comparison-based, whereas domains involving procedural or risk-related care more frequently incorporated verification and planning-oriented structures. This variation indicates that the configuration and unfolding of reasoning structures were closely related to the clinical domain in focus, rather than representing a single generalised reasoning pattern. Previous research has also described clinical reasoning as non-linear, often emphasising iterative or cyclical processes of interpretation and action (Tanner, 2006; Levett-Jones, et al., 2010), at times conflating the structural configuration of articulated reasoning with assumptions that RNs’ cognition itself is inherently non-linear.

In this study, we instead refer specifically to the structural properties of the coded reasoning structures and operator configurations in RNs’ reasoning. Although observation was commonly identified as the starting point of their reasoning structures, their accounts simultaneously suggest that engagement in reasoning could occur through any of several identified cognitive operators, e.g. verification or planning. This suggests that observation should not be understood as a fixed entry point, but rather as one of several possible points of engagement in reasoning, depending on the clinical domain and situational demands. Although research and literature have acknowledged this iterative dimension of clinical reasoning before (Levett-Jones et al., 2010; Simmons, 2010), and while established models have been critiqued for potentially simplifying the complexity of reasoning processes, these models remain widely used in research and education and continue to make important contributions to the understanding, teaching, and assessment of clinical reasoning (Calcagni et al., 2023; Doyon and Raymond, 2025). The consistent non-linearity and multiple starting points identified in this study suggest that clinical reasoning in home-based care may involve reasoning structures that are not always readily captured by phase-based conceptualisations of reasoning. Although the present study did not examine underlying cognitive mechanisms directly, the identified reasoning structures show some similarities to descriptions of clinical reasoning found in broader nursing and health-professions literature. For example, the movement between observation, evaluation, verification, and planning may be understood as consistent with previous discussions of iterative reasoning processes, dual-process perspectives, and abductive forms of clinical inference (Croskerry, 2009; Mirza et al., 2014; Nibbelink and Brewer, 2018). However, the present findings concern the structural organisation of retrospectively articulated reasoning rather than the cognitive processes themselves. Importantly, this study contributes to making part of RNs’ clinical reasoning less opaque and more accessible by showing how verbalised retrospective accounts can be broken down into traceable cues, operator assignments, and domain-specific reasoning structures. Through this approach we cannot claim to access RNs’ cognition directly, nor open the black box of RNs’ clinical reasoning; rather the approach offers a disciplined way of describing how reasoning is articulated in intricate, practice-near situations.

Future research should examine factors that may influence how clinical reasoning is structured and articulated in practice, including clinical experience, contextual knowledge, assumptions, metacognitive processes, and potential cognitive biases. Further research is also needed to explore how identified reasoning structures relate to clinical actions, decision quality, and patient outcomes, particularly in relation to safe, effective, and person-centred care.

Taken together, these findings suggest that clinical reasoning in home-based care may be better understood as a set of contextually situated and structurally variable reasoning structures, rather than as a linear or uniformly staged process. This has implications for both research and education, where prevailing models may risk oversimplifying how reasoning unfolds in practice. Recognising the patterned yet context-sensitive nature of reasoning structures may support the development of approaches that more accurately reflect the complexity of clinical work and enhance the ability to study, teach, and support clinical reasoning in practice.

## 5 Methodological considerations

The exploratory and descriptive qualitative design enabled in-depth, context-sensitive insight into RNs clinical reasoning as a complex phenomenon in clinical practice. To address the “black box” nature of cognitive processes, retrospective TA combined with the MCPA protocol were applied. Although retrospective TA has been criticised (van Someren et al., 1994), it provides access to reasoning in complex clinical situations as articulated by participants and accommodates practical and ethical constraints associated with concurrent approaches (Fonteyn et al., 1993). To mitigate recall bias, TA was conducted immediately following clinical situations and supported by stimulated recall, thereby reducing post hoc rationalisation. The findings should therefore be understood as structurally analysed retrospective verbal accounts anchored in observed care situations, rather than direct observations of real-time cognitive processes. The purposive sampling strategy ensured that participants had relevant clinical experience to address the study aim. The participants varied substantially in years of experience in home-based care. However, the study was not designed to examine relationships between experience level and clinical reasoning, and no comparative analyses based on years of experience were undertaken. In line with previous TA research, the focus was on information-rich cases rather than sample size (Lundgrén-Laine, et al., 2011).

The study incorporated AI-assisted analysis within a structured and rule-based analytic framework. While AI-supported qualitative analysis is an emerging and methodologically contested approach (Morgan, 2023; Turobov et al., 2024), it was applied under strict procedural control and in parallel with researcher-led analysis. The last author oversaw the AI-assisted procedures, while the first author conducted the manual analyses, allowing analytical processes to be performed independently and subsequently compared. This separation was intended to minimise mutual influence and strengthen analytical rigour. The MCPA protocol was therefore well suited, as it emphasises systematic and traceable handling of data. Consistent with previous research, AI was primarily applied to support data sorting, structured coding of cues, and identification of reasoning streams. The analytic workflow may also be understood as a methodological contribution, as it combines principles of protocol analysis with explicit rule systems for traceability, stream construction, and cross-case comparison. Further methodological work is needed to examine its applicability in other datasets and contexts.

The use of AI-assisted analysis warrants further consideration. While the analytical framework and interpretative responsibility remained with the research team, the AI-supported approach enabled a level of systematic pattern identification and cross-case comparison that would have been difficult to achieve through manual analysis alone. Rather than replacing researcher judgement, the AI functioned as an analytic support tool within a structured protocol, allowing for consistent application of coding procedures across transcripts. This approach facilitated the identification of recurring reasoning structures while maintaining transparency through an audit trail and continuous researcher validation. Simultaneously, the use of AI introduces methodological considerations related to interpretability and control, underscoring the importance of clearly defined analytic procedures, iterative testing, and researcher oversight. The AI-assisted components did not function as an interpretive agent. Their role was restricted to supporting the application of the rule-based analytic protocol (MCPA) under researcher control. No themes, explanatory categories, or conceptual abstractions were generated by the AI.

Rigour was supported through predefined analytic rules, iterative testing of protocol instructions, and maintenance of an audit trail. A subset of transcripts (n = 10) was analysed manually and compared with AI-assisted outputs to ensure consistency and adherence to the MCPA protocol. In addition, the remaining authors acted as continuous critical reviewers throughout the analytic process and conducted independent manual checks of selected transcripts, providing an additional layer of analytical validation. The findings are context-bound to home-based nursing care and should be interpreted accordingly; however, the pipeline-based structured analytic approach and focus on clinical reasoning processes may support transferability to similar clinical contexts.

## 6 Implications and conclusions

The findings may have practical implications for nursing education, clinical supervision, and professional development. By making reasoning structures more explicit and traceable, the proposed approach may support reflection on clinical reasoning, facilitate discussion of complex care situations, and provide a language for examining how reasoning unfolds across different clinical domains. In conclusion, this study shows that RNs’ clinical reasoning in home-based care can be described as patterned, multi-layered, and contextually sensitive. The identified variation across clinical domains and the consistent non-linear organisation of reasoning structures highlight the limitations of linear models of reasoning. The proposed analytic approach provides a structured and transparent way of examining how reasoning is articulated in practice, offering a foundation for further research into complex cognitive processes in nursing. These findings may also support the use of structured approaches to make clinical reasoning visible in nursing education and clinical supervision, enabling more explicit discussion and development of reasoning in practice.

## Disclosure

This research report is part of the PhD project DECIDE (Developing Expertise in Care and Informed Decision-making), which is included in the Nordic collaboration and the programmatic research platform Continuity for Quality of Care in Nursing (CARE) at Lovisenberg Diaconal University College, Norway, and Karlstad University, Sweden. The DECIDE project employs a multi-method design focusing on nurses’ scope of practice (SoP), including their decision-making processes and the factors that influence them—primarily in the context of home-based care. The project uses functional ability and frailty among older adults as clinical examples of conditions that contribute to complex care needs. DECIDE is being conducted between 2021 and 2026.

Prior to submission, a GDPR-compliant version of ChatGPT was used to assist with readability, language refinement, and sentence structure. The tool was used solely during the writing process to optimize the final draft, including after revisions of the manuscript. On both occasions, the authors critically reviewed and edited all content to ensure that the AI's contributions were limited to minor language improvements and did not affect the substance of the work. The authors take full responsibility for the content of the published article.

## Funding

No external funding.

## CRediT authorship contribution statement

**Ida Røed Flyum:** Writing – original draft, Methodology, Investigation, Formal analysis, Data curation, Conceptualization. **Veronica Pavedahl:** Writing – review & editing, Supervision, Methodology, Formal analysis, Conceptualization. **Anna Josse Eklund:** Writing – review & editing, Supervision, Methodology, Formal analysis, Conceptualization. **Gunilla Borglin:** Writing – review & editing, Supervision, Methodology, Investigation, Formal analysis, Conceptualization.

## Declaration of competing interest

The authors declare that they have no known competing financial interests or personal relationships that could have appeared to influence the work reported in this paper.

## Data Availability

The raw data that supports the findings of this study are not available due to confidentiality and ethical restrictions. The retrospective TA guide are available upon reasonable request from the corresponding author.
